# Detection of Surface and Subsurface Cracks in Metallic and Non-Metallic Materials Using a Complementary Split-Ring Resonator

**DOI:** 10.3390/s141019354

**Published:** 2014-10-16

**Authors:** Ali Albishi, Omar M. Ramahi

**Affiliations:** Electrical and Computer Engineering Department, University of Waterloo, 200 University Avenue West, Waterloo, ON N2L 3G1, Canada; E-Mail: aalbishi@KSU.EDU.SA

**Keywords:** crack detection, complementary split-ring resonator (CSRR), microstrip line technology, near-field detection, metamaterials

## Abstract

Available microwave techniques for crack detection have some challenges, such as design complexity and working at a high frequency. These challenges make the sensing apparatus design complex and relatively very expensive. This paper presents a simple method for surface and subsurface crack detection in metallic and non-metallic materials based on complementary split-ring resonators (CSRRs). A CSRR sensor can be patterned on the ground plane of a microstrip line and fabricated using printed circuit board technology. Compared to available microwave techniques for sub-millimeter crack detection, the methods presented here show distinct advantages, such as high spatial resolution, high sensitivity and design simplicity. The response of the CSRR as a sensor for crack detection is studied and analysed numerically. Experimental validations are also presented.

## 1. Introduction

The responses of materials to electrodynamic fields depend on their electrical properties, such as permittivity, permeability and conductivity. Characterising such properties for the purpose of detecting changes in material is important in many areas, such as the food industry, bio-sensing applications and surface and subsurface crack detections, to name but a few [1–7]. Material defects, such as sub-millimeter cracks in metallic and non-metallic materials, affect the near-field distribution of the electric and magnetic fields. Such a change in the field distribution (magnitude and phase) is a consequence of change in the medium effective constitutive parameters. These changes can be used as signatures for crack detection purposes, which is the underlying physics behind the detection of cracks in metallic and non-metallic media in general.

Inspection for surface and subsurface cracks in metallic and non-metallic materials is critical for quality assessment and maintenance. Microwave techniques have been proposed and utilized for crack detection. In [8], an open-ended waveguide operating at around 20 GHz was used as a microwave technique for crack detection in metallic materials, where a 840 *μ*m wide surface crack was detected by direct correlation with the change in the reflection coefficient. While capable of detecting sub-millimeter cracks, the waveguide gives rise to a small perturbation in the near field, leading to small changes in the reflection coefficient; thus, the demand for higher accuracy was needed [9]. In [10–12], methods were introduced to increase the sensitivity of open-waveguide sensors. In [9], the detection of micro-scale cracks measuring 200 *μ*m in width in steel plates were studied using near-field microwave dual-behavior resonator (DBR) filters, where a frequency shift in the resonance frequency indicated the presence of a crack. In the DBR technique, two filter probes were used with operating frequencies of 13.577 GHz and 8.008 GHz. The sensor exhibited resonance frequency shifts between 39 and 51 MHz. However, the changes in the resonance frequencies were small, indicating low sensitivity. In addition, the minimum transmission and reflection coefficient (indicative of the presence of a crack) was spread over a relatively wide band, which made finding the resonance frequency difficult, especially if the sensor were to be integrated in a stand-alone detection system rather than a high precision spectrum analyzer.

Electromagnetic fields in the microwave regime can penetrate and interact with dielectric material, provided that the permittivity of the media is not excessively high. The result of the interaction can be used in many applications, such as detecting sub-millimeter cracks in dielectric material. Several microwaves techniques for crack detection in non-metallic material have been reported. In [13], an open-ended waveguide antenna was used to detect flaws (holes) ranging in diameters from 0.025 and 0.59 mm in ceramic materials (*Si*_4_*N*_4_) used in fabricating gas turbines. Since the technique in [13] is based on the far field, which is based on reflection and refraction, the perturbation in the size of the defects or anomalies in the material under test needs to be proportional to the wavelength, thus requiring operation in the W band (75 GHz to 110 GHz). Such operation frequency makes the measurement setup expensive and complex. To overcome this problem, microwave near-field techniques were introduced to detect cracks in non-metallic materials. Near-field techniques are dependent on the evanescent type of fields having effective wavelengths much higher than the wavelength in free space, which makes detection of particles having sub-wavelength features possible. In [12], an open-ended waveguide, similar to the one reported in [8], was used to detect cracks in cement-based materials where the basic detection principle is the complex interaction between the near field at the end of the waveguide and the material under test. To the best knowledge of the authors and based on the available literature, the available detection techniques for crack detection in metallic and non-metallic materials have at least one of the following challenges: (1) working at a relatively high frequency; (2) requiring a complex measurement setup; (3) a relatively high manufacturing cost; (4) low sensitivity; and (5) low resolution.

In [7], a method for sub-millimeter surface cracks in metallic material based on the near-field microscopy technique using a complementary split-ring resonator (CSRR) was introduced for the first time and was validated experimentally. The technique used a CSRR as a sensor, which was able to detect sub-millimeter surface cracks with a resolution of $\frac{\lambda }{600}$ and to resolve two adjacent cracks separated by 1 mm. A recent paper by Taehwa *et al.* [14] used the same CSRR reported in [7] for crack detection in metallic material, but with a substrate integrated waveguide (SIW) for excitation.

In this paper, we study CSRR sensors for crack detection in metallic and non-metallic materials. We introduce design formulae that can be used by engineers and scientists to easily design CSRR sensors and predict their resonance frequencies. We then investigate inexpensive techniques to increase the sensitivity of CSRR sensors and to decrease the operating frequency. We focus on the surface current distribution as the underlying physical mechanism behind the CSRR sensor. We consider the effect of the geometrical features on the CSRR resonance frequency with the aim of achieving an overall small form factor. Finally, we show that the CSRR sensor is effective, not only for sensing cracks in metallic surfaces, but also non-metallic material, such as fiberglass, plastics, carbon fibers, amongst other dielectric materials that are increasingly used in aircraft structures and high performance applications [15].

## 2. Complementary Split-Ring Resonator as a Near-Field Sensor

Quasi-static resonators are designed such that the resonators' structures are electrically small compared with the electromagnetic field wavelength of the excitation source. The resonance phenomenon in quasi-static resonators is based on the effective tightly-distributed capacitance and inductance of the structure, unlike resonators of dimensions comparable to the wavelength, where the resonance phenomenon is based on phase propagation. Several works provided circuit models for CSRRs [16–18]. Generally, the resonance frequency can be formulated as.

(1) $$f_{0}=\frac{1}{2\pi \sqrt{L_{T}C_{T}}}$$

where *L_T_* and *C_T_* are the total distributed inductance and capacitance in the structure, respectively. There have been many designs used to realize quasi-static resonators, where loops create current circulations that directly contribute to the effective inductance and gaps affect the net capacitances. Examples include split-ring resonators (SRRs) [19], spiral resonators [20] and Hilbert curves [21]. Like SRRs, CSRRs are quasi-static resonators that were originally conceived of based on Babinet's principle. The CSRRs can be designed by creating the complement of the SRR, whereby copper is replaced by air and *vice versa*. Strictly speaking, the CSRR etched on a substrate is semi-complementary to the SRR due to the presence of the dielectric substrate essential for the construction of the sensor.

At the resonance frequency of a resonator, the electric and magnetic energy densities are enhanced significantly at certain locations in the close proximity of the resonator [19]. Any disturbance of the electromagnetic fields around the resonator causes the resonance frequency to shift. The shift in the resonance frequency of any resonator and the electrical properties of a material under test (MUT), such as the permittivity and permeability, can be related to each other according to [22]:

(2) $$\frac{\Delta f_{r}}{f_{r}}=\frac{\int_{\upsilon }(\Delta \varepsilon {\text{E}}_{1}\cdot {\text{E}}_{0}+\Delta \mu {\text{H}}_{1}\cdot {\text{H}}_{0})d\upsilon }{\int_{\upsilon }(\varepsilon_{0}{\left|E_{0}\right|}^{2}+\mu_{0}{\left|H_{0}\right|}^{2})d\upsilon }$$

where Δ*f_r_* is the shift in the resonance frequency, *f_r_*, Δϵ and Δ*μ* are the change in the permittivity and permeability and υ is the perturbed volume. **E**_0_ and **H**_0_ are the field distributions without the perturbation and **E**_1_ and **H**_1_ are the field distributions with the perturbation. If the perturbation is small and the field distribution is assumed unchanged, then Equation (2) reduces to:

(3) $$\frac{\Delta f_{r}}{f_{r}}=\frac{\int_{\upsilon }(\Delta \varepsilon {\left|E_{0}\right|}^{2}+\Delta \mu {\left|H_{0}\right|}^{2})d\upsilon }{\int_{\upsilon }(\varepsilon_{0}{\left|E_{0}\right|}^{2}+\mu_{0}{\left|H_{0}\right|}^{2})d\upsilon }.$$

The perturbation approach is used in resonant cavity methods and near-field sensors to extract the constitutive properties of materials (permittivity and permeability) [23].

**Figure 1.**
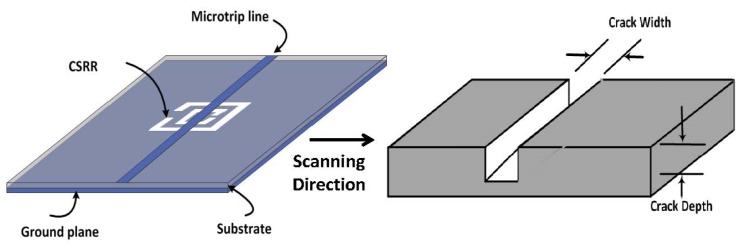
A schematic of a CSRR excited by a microstrip line used as a near-field sensor to detect defects in metallic surfaces.

**Figure 2.**
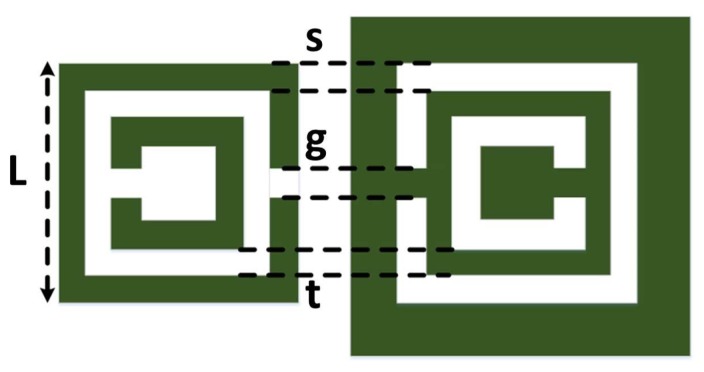
The geometry of a CSRR and SRR, where *L* = 3 mm, *g* = 0.2 mm, *s* = 0.2 mm and *t* = 0.2 mm.

When the electrodynamic near field of a resonator is disturbed due to the presence of either new material or deformation (such as topological anomalies) in the original material, the resonance frequency exhibits a shift that can be used as a signature for detection or for characterizing the new environment in general. In this paper, we used CSRR excited by a microstrip line as a near-field sensor. Figure 1 shows a CSRR sensor that is used to detect defects in metallic surfaces, such as cracks. The CSRR sensor is a planar structure excited by a microstrip line, which produces an electric field perpendicular to the surface of the sensor. The CSRR sensor has a conducting center island surrounded by a conducting plane that connects the center island through two narrow bridges, as shown in Figure 2.

The primary objective when designing a sensor is to obtain a high measurement sensitivity, reduce measurement setup complexity and use widely available relatively inexpensive microwave components. Since the near field of any electromagnetic source occupies a space directly proportional to the size and frequency of the radiator, to effectively sense small changes, such as sub-millimeter cracks in materials in the near field of the sensor, the overall size of the CSRR must be sufficiently small so as not to be much larger than the features to be detected. In the examples we considered here, the gap separations and trace widths of the sensor were chosen to be 0.2 mm as the smallest feature in this design. Generally, there is flexibility in designing a CSRR sensor at a wide range of frequencies associated with a target crack size.

## 3. CSRR Sensor Design Approach

Unlike conventional wire antennas, where the resonant frequency is directly proportional to the length of the radiator, the resonant frequency of electrically-small resonators, such as CSRR and SRR resonators, is typically found through measurement or simulation [7,16,18,24,25]. In general, however, the larger the overall size of the electrically-small resonator, the lower its resonant frequency Here, we consider correlating the topological dimensions of a CSRR element to its resonant frequency for the purpose of obtaining useful design guidelines. Because the sensor is a two-port microstrip line exciting a CSRR, a vector network analyzer (VNA) is used to measure the reflection and transmission parameters. In order to match the internal impedance of the VNA in our measurement setup, the microstrip line was designed to have a characteristic impedance of 50 Ω (the microstrip line can be designed to match the internal impedance of other measurement setup devices). To achieve a 50 Ω characteristic impedance for the microstrip line using Rogers RO4350 substrate with a thickness of 0.75 mm, a permittivity of 3.66 and a loss tangent of 0.0031, the width of the microstrip line was 1.7 mm.

**Figure 3.**
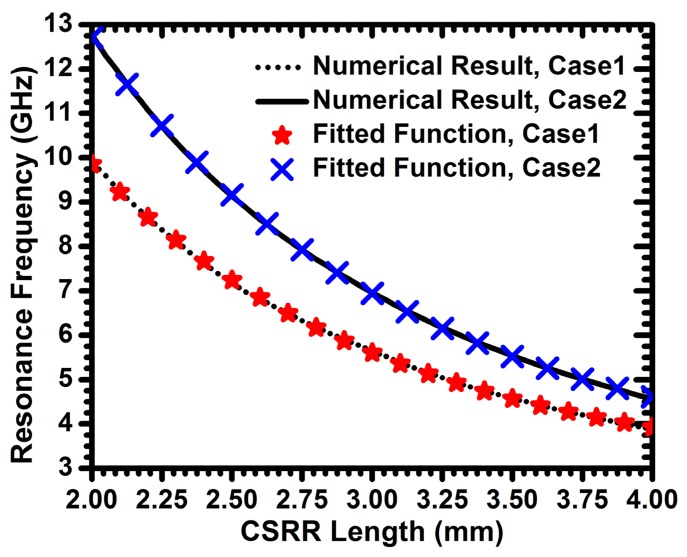
Resonance frequencies *versus* CSRR length obtained form full-wave numerical simulation and from the fitting exponential function for gap separations and trace widths of 0.1 mm and 0.2 mm labeled as Case 1 and Case 2, respectively.

For the purpose of developing CSRR sensor design guidelines, the CSRR length was varied from 2 to 4 mm. Two different values of 0.1 mm and 0.2 mm were considered for the gap separations and trace widths of the structure. The resonance frequencies *versus* the size of CSRRs (considered to be its length) are plotted in Figure 3. An exponential fitting function was used to find the resonance frequency of a CSRR as a function of its length, expressed as:

(4) $$f_{r}=e^{a+bL+cL^{2}}$$

where Lis the length of the resonator and a, b and c are the fitting function constants. For gap separations and trace widths of 0.1 mm, the constants were *a* = 4.041, *b* = −1.085 and *c* = 0.104. In the case of the gap separations and trace widths of 0.2 mm, the constants were *a* = 4.34, b = −1.093 and *c* = 0.098.

Figure 3 shows that the resonance frequencies decrease exponentially by increasing the overall lengths of the resonator. Of course, lowering of the resonant frequency is expected due to the increase in the effective inductance of the SCSR; however, the exponential relationship could not have been intuitively predicted.

**Figure 4.**
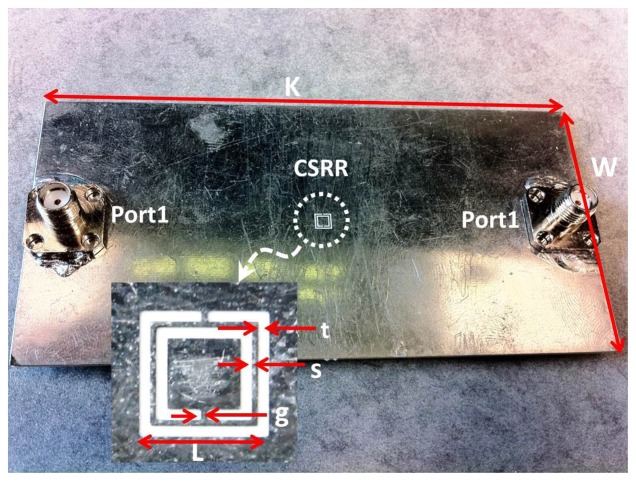
The CSRR printed on the ground plane of a microstrip line with its fabricated dimensions, *L* = 0.27 mm, *g* = *s* = *t* = 0.16 mm, *K* = 100 mm and *W* = 50 mm.

A CSRR sensor was designed to resonate at around 6.95 GHz with resonator dimensions of *L* = 3 mm and *s* = *g* = *t* = 0.2 mm (see Figure 2). For the purpose of crack detection, designing a sensor to operate at a specific resonance frequency is unnecessary as long as the resonance frequency and dimensions of the sensor are chosen to detect cracks with dimensions falling within a specific range. Figure 4 shows a photo of the designed CSRR with dimensions of *L* = 0.27 mm and *g* = *s* = *t* = 0.16 mm.

**Figure 5.**
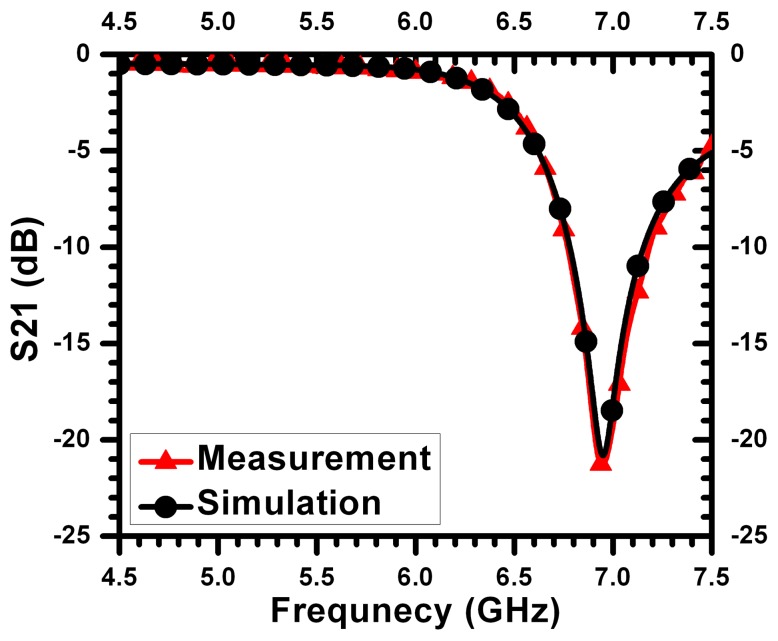
Frequency response of the sensor obtained from full-wave simulation using HFSSand measurements for a 3-mm sensor.

Figure 5 shows a comparison between the simulation and measurements for a sensor with *L* = 0.27 mm and *g* = *s* = *t* = 0.16 mm. The feeding microstrip line had a width of 1.7 mm on Rogers RO4350 substrate with a thickness of 0.75 mm, permittivity of 3.7 and a loss tangent of 0.003. The agreement between the full-wave simulation and measurements is observed to be very strong, especially at the frequency of minimum transmission. This gives strong confidence in the capability of numerical simulation to determine the response of different sensors and different crack topologies.

## 4. Sensing Mechanism in CSRR Sensors

**Figure 6.**
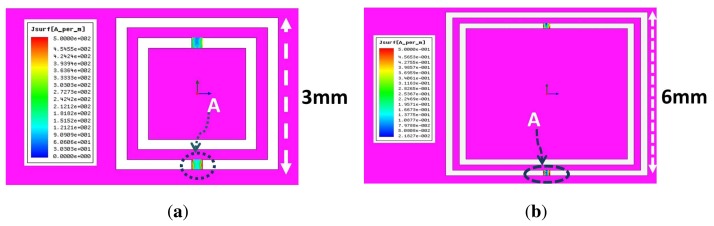
Current distribution density on CSRRs at the resonance frequency. The bridge labeled A shows the area of high current density. (**a**) A CSRR of a length of 3 mm. (**b**) A CSRR of a length of 6 mm. The surface current on the 3-mm CSRR is one order of magnitude higher than that of the 6-mm CSRR.

**Figure 7.**
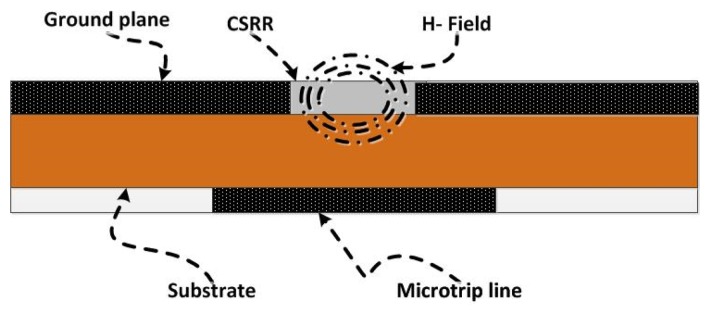
Magnetic field lines around the bridge line labeled A in Figure 11.

The sensitivity of the CSRR sensor is determined by how much the near field of the resonator can be disturbed by defects in materials causing the resonance frequency to shift. In addition, the size of detects is essential for determining the length of the CSRR, which, in turn, corresponds to the resonance frequency of the sensor. In this section, we investigate factors affecting the sensitivity of the CSRR sensor by careful analysis of the surface current distribution on the resonator. We considered two CSRRs of gap separations and trace widths of 0:2 mm with different lengths of 3 mm and 6 mm. Figure 6 shows the current surface distribution density on the narrow bridges, which was found to be the highest over the entire sensor CSRR surface. The maximum surface current density yields the highest magnetic field from any part of the sensor, and thus, the most sensitive part of the CSRR sensor is the narrow bridge linking the inner island to the outer plane conductor. Figure 7 shows the magnetic field lines around the sensing element.

**Figure 8.**
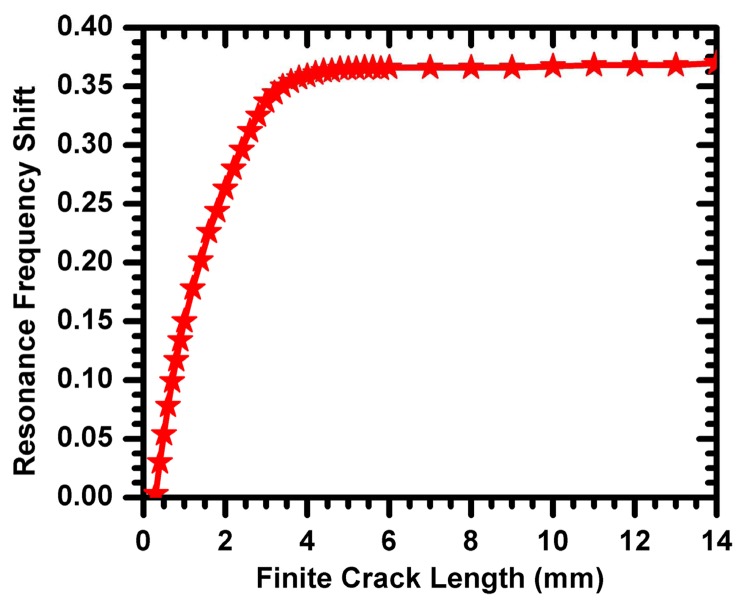
Resonance frequency shift as a function of the crack length. The crack is 0.2 mm wide and 2 mm deep.

**Figure 9.**
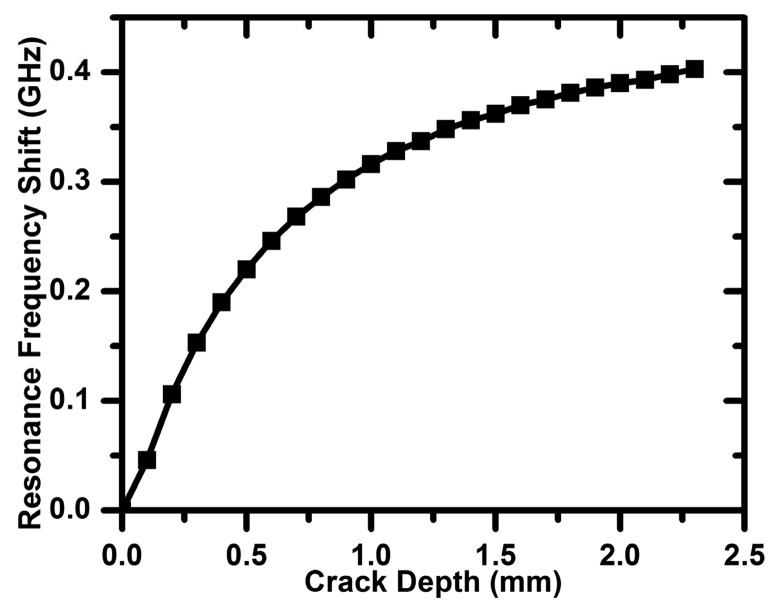
Resonance frequency shift as a function of the crack depth. The crack is 0.2 mm wide and 10 mm in length.

The resonance frequency of the CSRR sensor (plotted in Figure 3) represents the sensor in the absence of the metallic surface to be detected. When the sensor is placed on the solid metallic surface, the resonance frequency shifts. The resonance frequency of the sensor in the presence of the solid crack-free surface is used as the reference resonance frequency. Any shift in the resonance frequency due to the presence of a crack is then compared to the reference frequency. The crack considered in [7] extended across the entire width of the aluminum block under test. Here, we use numerical simulation to test the effectiveness of the CSRR sensor to detect cracks of finite length, which represents a scenario more practical than that of very long cracks. Figure 8 shows the resonance frequency shift experienced by the 3-mm CSRR sensor for different crack lengths (a crack width of 0.2 mm and a depth of 2 mm). For a crack with a very short length, Figure 8 shows a very small shift in the resonance frequency, as would be expected. As the crack length increases, the shift in the resonance frequency increases, until the crack length becomes approximately equal to the length of the sensor (in this example, a 3-mm CSRR sensor was used). Increasing the crack length further did not lead to any additional increase in the resonance frequency shift. In Figure 9, we show the effect of varying the crack depth on the resonance frequency shift for a 0.2 mm-wide and 15 mm-long crack.

## 5. Increasing the Sensitivity of CSRR Sensors

**Figure 10.**
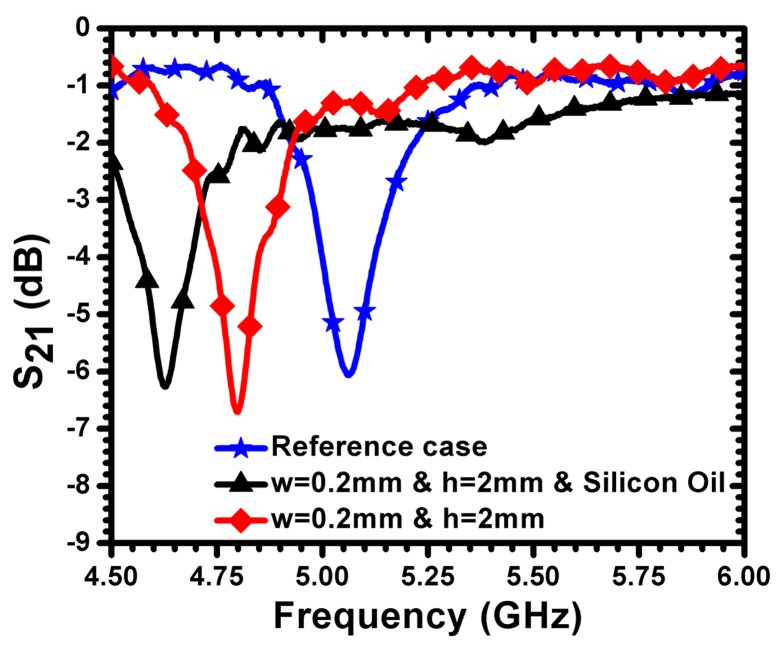
Minimum transmission of the sensor for a crack width of 200 *μ*m and a depth of 2 mm for the reference case (without a crack) and for the cases of a crack with and without silicon oil.

Defects in metallic surfaces, such as sub-millimeter cracks, introduce capacitance that is parallel to the effective capacitance of the sensor. The crack-induced capacitance leads to a downward shift in the resonance frequency in accordance with Equation (1). Therefore, the sensitivity of the sensor can be enhanced by increasing the capacitances of the crack by filling the crack with liquid dielectric material. For this purpose, a crack was filled with silicon oil of a dielectric constant of 2.70. The test sample was fabricated using an aluminum plate with a crack width of 200 μm and a depth of 2 mm. Figure 10 shows the measured frequency of minimum transmission coefficient for the reference case (solid aluminum surface without a crack) and for the cases of the crack with and without silicon oil. The filling of the crack with silicon increased the sensitivity of the sensor by 150 MHz (a shift of more than 435 MHz was registered with respect to the case of solid aluminum without the crack). It is expected that liquids with higher dielectric constants can increase the sensitivity further. In practical crack detection scenarios, silicon oil or other liquids can be applied to the surface of the metallic material to be detected prior to the sensing procedure.

## 6. CSRR Miniaturization Using Lumped and Distributed Elements

Near-field sensors operating at lower frequency are relatively larger than sensors operating at higher frequencies. The sensitivity of relatively large sensors to detect small defects in materials, such as sub-millimeter cracks, becomes lower in comparison to smaller sensors. Sensors with a small form factor are more advantageous than larger sensors, since they are easier and less expensive to integrate in practical stand-alone sensing systems. Therefore, miniaturization of sensors, while maintaining a relatively low resonant frequency, has clear advantages.

**Figure 11.**
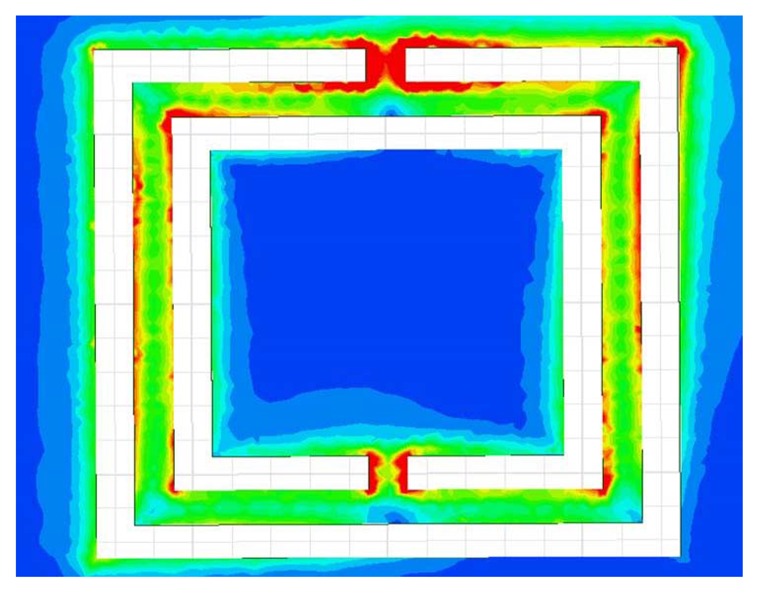
Simulated current surface distribution on a 3-mm CSRR at the resonance frequency.

One immediate choice for miniaturization of CSRR sensors is the introduction of lumped inductance in the path of the dominant surface current. By understanding the surface current distribution on the surface of the CSRR, a lumped inductor can be placed at the location of the highest current. Figure 11 shows the current distribution at the resonance frequency of a CSRR of a length of 3 mm. Clearly, the current surface distribution is highly concentrated at the two narrow bridges, indicating that the magnetic field of the sensor is highest around the two bridges, as discussed above. Replacing the narrow bridges by a lumped inductor adds additional inductance, which results in shifting the resonance frequency downward.

**Figure 12.**
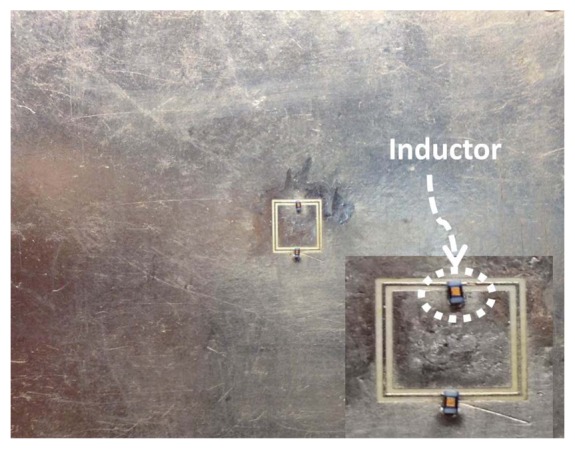
A CSRR after replacing the two narrow conducting bridges with lumped inductors. The red color indicates the highest current magnitude.

**Figure 13.**
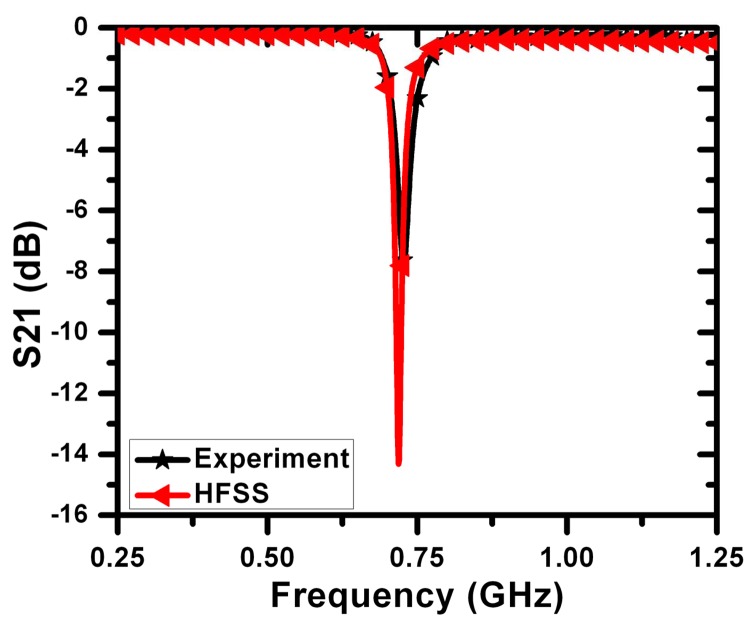
Minimum transmission coefficient of the sensor obtained from HFSS and measurement of 6-mm CSRR with respect to the case without the crack after adding the two inductors of a value of 20 nH.

**Figure 14.**
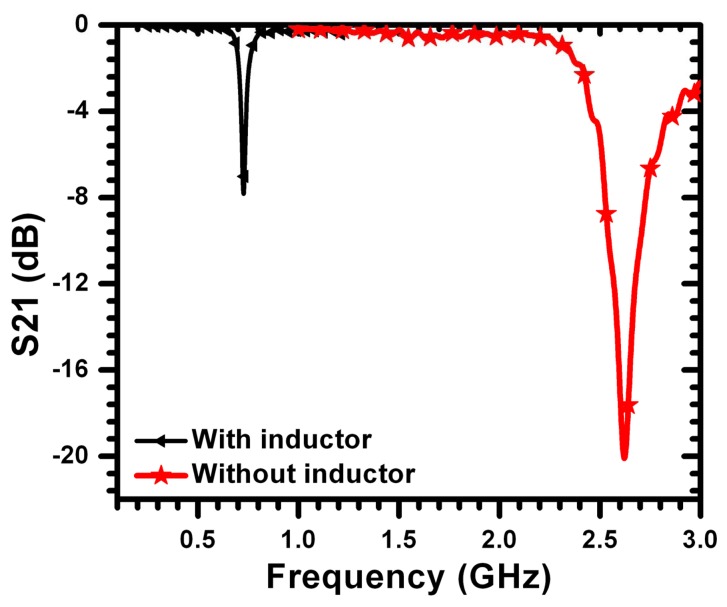
Minimum transmission coefficient of the sensor obtained from measurement with and without inductors of 6-mm CSRR.

To test the effectiveness of the added lumped inductor on the sensitivity of the CSRR sensor, a CSRR of a length of 6 mm was designed using full-wave numerical simulation and tested experimentally. Then, the narrow bridges were replaced by two 20 nH inductors, as shown in Figure 12. Figure 13 shows the minimum transmission coefficient of the CSRR sensor with the lumped inductors. A strong agreement between the measurement and simulation is observed. Figure 14 shows the experimental result of the CSRR before and after replacing the narrow bridges. We observe almost a reduction of the resonance frequency by a factor of five. Using numerical simulation, the miniaturization, however, was not found to yield sufficient sensitivity for detecting the types of cracks considered above (the resonance frequency exhibited a very small shift, not shown here for brevity). Therefore, while the introduction of lumped elements indeed achieved miniaturization, the sensitivity of the sensor decreased due primarily to the high localization or confinement of the magnetic field within the added inductors.

**Figure 15.**
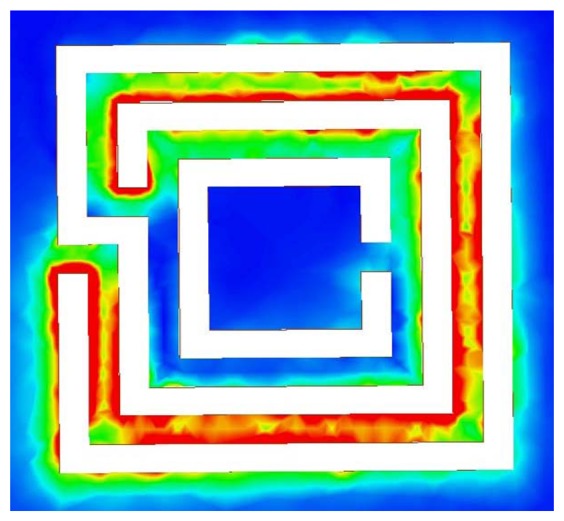
Simulated current distribution on a new CSRR at a resonance frequency of around 3.781 GHz. The red color indicates the highest current magnitude.

**Figure 16.**
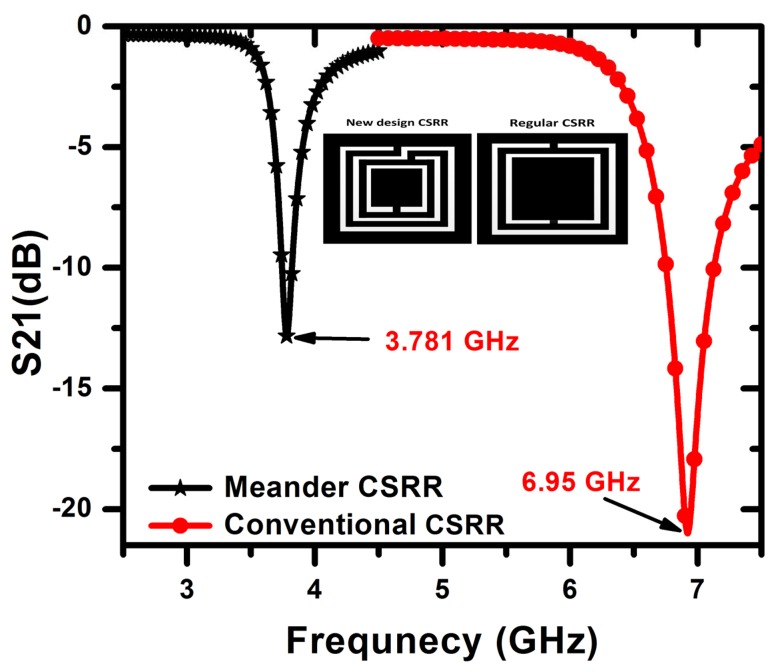
Minimum transmission coefficient of the two sensor obtained from HFSS at the resonance frequency of the 3-mm CSRR.

**Figure 17.**
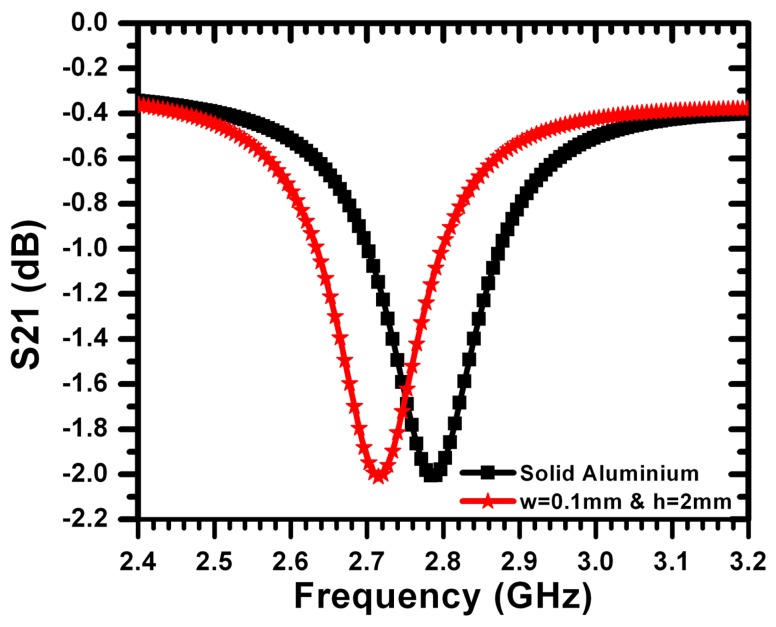
Minimum transmission of the new CSRR sensor for a crack width (w) of 100 *μ*m, and the crack depth (h) is 2 mm compared with solid aluminium.

Instead of using lumped inductors, inductance can be added to the CSRR sensor by employing a different design involving meander lines, as shown in Figure 15. Figure 15 depicts the current distribution at the resonance frequency of the CSRR sensor. Figure 16 shows the transmission coefficient of the CSRR and meandering CSRR. The meandering increased the total inductance, such that the resonance frequency shifted downward by approximately 50% with respect to the original CSRR design response. Figure 17 shows that for a long crack with a width of 100 *μ*m and a depth of 2 mm, the meander CSRR sensor resulted in a frequency shift of more than 70 MHz. Therefore, we conclude that increasing the total inductance of a CSRR for miniaturizing purposes using either lumped or distributed elements is possible; however, the sensitivity of the CSRR is decreased for crack detection purposes, but the sensor has the advantage of achieving resonance at a lower frequency.

## 7. Crack Detection in Non-Metallic Materials

**Figure 18.**
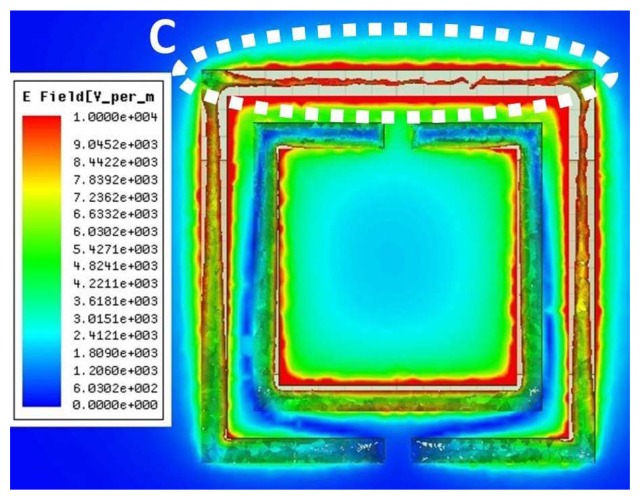
Total electric field distribution on a CSRR at the resonance frequency of a 3-mm CSRR. The red color indicates the highest current magnitude.

Detection of anomalies, such as cracks in dielectric material, is based on a change in the electric field due to the presence of the anomaly. Therefore, the sensing element for the near-field detection of anomalies, such as cracks in dielectric surfaces, needs to have a dominant electric field. By investigating the total electric field on the surface of the CSRR sensor, we observed a dominant electric field along one side of the CSRR. Figure 18 shows the maximum electric field distribution on a 3-mm CSRR at the resonance frequency. The sensing element for the purpose of detecting dielectric anomalies, therefore, can be considered as the side depicted by the letter C in Figure 18.

**Figure 19.**
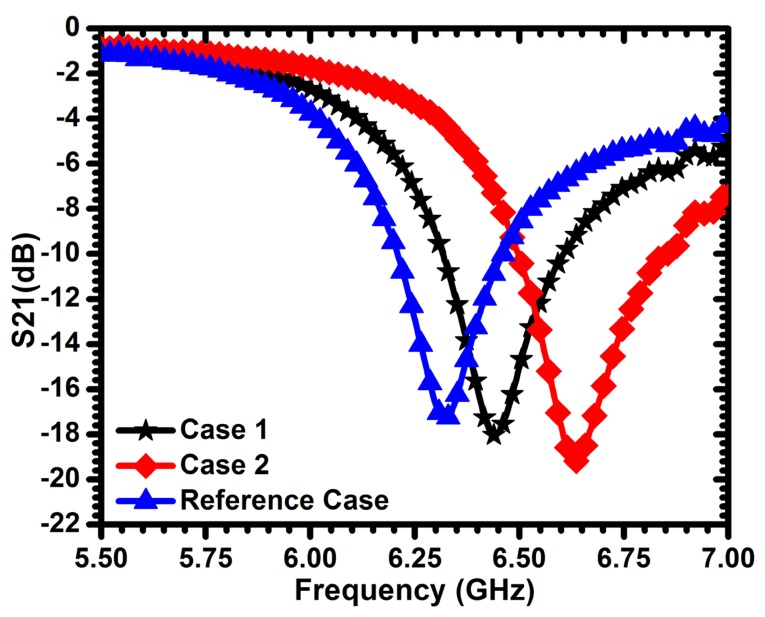
Minimum transmission of the sensor for a crack width (w) of 150 *μ*m (Case 1) and 200 *μ*m (Case 2) compared with solid dielectric material (fiberglass) with a dielectric constant of 2.3 (reference case). The crack depth (h) is 1.5 mm.

**Figure 20.**
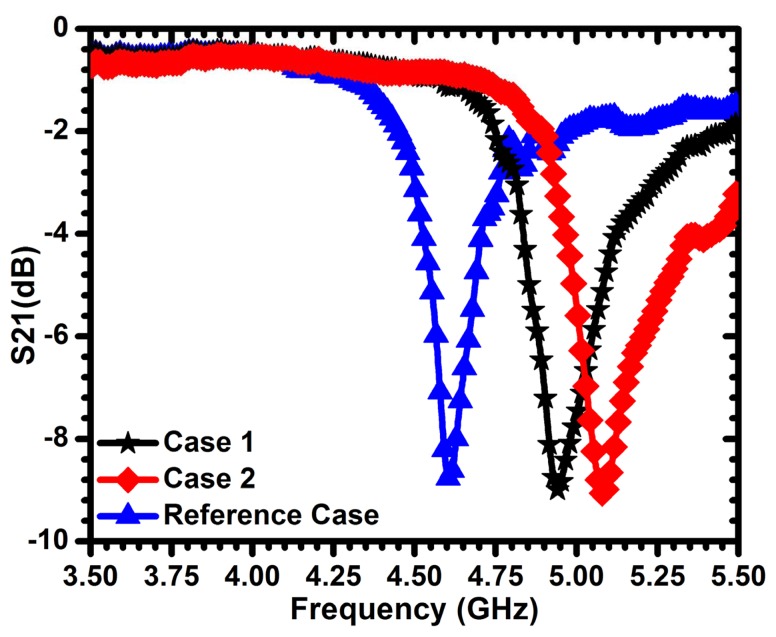
Minimum transmission of the sensor for a crack width (w) of 150 *μ*m (Case 1) and 200 *μ*m (Case 2) compared with a solid dielectric material, ceramic (ceramic material), with a dielectric constant of 9.8 (reference case). The crack depth (h) is 1.5 mm.

For validation purpose, a CSRR of a length of 3 mm is used to detect sum-millimeter cracks in dielectric materials. Fiberglass and ceramic materials with dielectric constants of 2.3 and 9.8, respectively, were used. Figure 19 shows the frequency of minimum transmission coefficient obtained experimentally when the sensor passes over a fiberglass material with different crack widths of 150 *μ*m and 200 *μ*m (depths of 1.5 mm). A maximum shift of more than 312 MHz was observed with respect to the reference case. For the ceramic material considered, Figure 20 shows a maximum shift of more than 477 MHz for the two crack topologies considered. These results indicate that the sensitivity increases with the dielectric constant of the material under test. Based on Figures 19 and 20, we observe that the cracks result in an upward shift in the resonance frequency (in contrast to the downward shift observed in the case of detecting cracks in metallic surfaces), which is consistent with the prediction of Equation (1), due to the decreased capacitance experienced by the sensor.

## 8. Conclusions

This work presented a comprehensive analysis of the capabilities of the CSRR sensor to detect sub-millimeter cracks in metallic and non-metallic surfaces. Design formulae were derived based on full-wave numerical simulation to provide the designer with guidelines that can eliminate expensive full-wave numerical simulations. Analysis of the surface currents on the CSRR revealed important clues as to the active sensing element that is most sensitive to changes in the surface under test. Miniaturization of the CSRR sensor was studied using two approaches using lumped elements and meandering. The lumped element approach achieved miniaturization, but did not yield any appreciable sensitivity, while the meandering achieved a reduction in the form factor of the sensor by 50% while achieving sufficient sensitivity for the detection of sub-millimeter cracks. Based on the physics of the CSRR sensing mechanism, a dielectric liquid, such as silicon oil, was introduced to enhance the sensitivity of the sensor by approximately 150 MHz for the case considered. Finally, the CSRR sensor was also found to be highly effective for detecting sub-millimeter cracks in dielectric surfaces.
